# Proceedings of cell-free noncoding RNA biomarker studies in liquid biopsy

**DOI:** 10.1515/rir-2025-0018

**Published:** 2025-10-04

**Authors:** Yumin Zhu, Fengping Wu, Kangping Liu, Shaozhen Xing, Chun Ning, Meng Ning, Heyue Jin, Yun Shao, Zhenye Zhu, Hongke Wang, Binbin Shi, Yajin Mo, Xinping Tian, Mengtao Li, Jiuliang Zhao, Zhi John Lu, Ning Gu

**Affiliations:** Medical School, Nanjing University, Nanjing 210093, Jiangsu Province, China; Nanjing Key Laboratory for Cardiovascular Information and Health Engineering Medicine, Institute of Clinical Medicine, Nanjing Drum Tower Hospital, Medical School, Nanjing University, Nanjing 210093, Jiangsu Province, China; Department of Maternal & Child and Adolescent Health, School of Public Health, Anhui Medical University, Hefei 230032, Anhui Province, China; MOE Key Laboratory of Bioinformatics, State Key Lab of Green Biomanufacturing, Center for Synthetic and Systems Biology, School of Life Sciences, Tsinghua University, Beijing 100084, China; Institute for Precision Medicine, Tsinghua University, Beijing 100084, China; Department of Rheumatology and Clinical Immunology, Peking Union Medical College Hospital, Chinese Academy of Medical Sciences, Peking Union Medical College, National Clinical Research Center for Dermatologic and Immunologic Diseases (NCRC-DID), Ministry of Science & Technology State Key Laboratory of Complex Severe and Rare Diseases, Ministry of Education Key Laboratory of Rheumatology and Clinical Immunology, Beijing 100730, China; Tianjin Third Central Hospital, Tianjin 300170, China; The Center for Regeneration Aging and Chronic Diseases, School of Basic Medical Sciences, Tsinghua University, Beijing 100084, China

**Keywords:** cell-free, noncoding RNA, liquid biopsy, transcriptome, disease biomarker

## Abstract

Liquid biopsy has great application value in the field of precision medicine because of its non-invasiveness, sensitivity and dynamics. Cell-free RNA molecules are one of the emerging biomarkers that can be used for liquid biopsy, and cell-free non-coding RNAs have become main RNA molecular markers because of their high abundance and stability, as well as their regulatory roles in basic development. It provides clues for the diagnosis, prognosis and monitoring of a variety of complex diseases, including rheumatic and immune diseases. This article describes the characterization of cell-free non-coding RNAs and bioinformatics strategies, and summarizes cell-free non-coding RNA biomarkers associated with rheumatic and immune diseases. Prospects and reflections are made on the further research and clinical application of cell-free RNA markers.

## Introduction

Cell-free RNA (cfRNA) was first discovered in plasma and serum in 1972. In 2006 and 2007, two groundbreaking works demonstrated that RNAs are present in microvesicles and exosomes, respectively, and that these RNAs can be secreted outside cells to act as signaling molecules to influence the behavior of recipient cells. This process can occur between adjacent cells and can be regulated over long distances.^[1,2]^ These two works opened the prelude for cfRNA research and brought new perspectives for exploring signal transduction between cells. Subsequently, cfRNAs were found to be present in almost all biological body fluids, including blood, saliva, urine, breast milk, cerebrospinal fluid, amniotic fluid, ascites, bile, and pleural effusion.^[3]^ The development of new technologies has created opportunities for the identification of cfRNA biomarkers. For example, a multinational research team led by Stanford University in the U. S. spent ten years developing RARE-seq, an optimized cfRNA detection method. This technology overcomes the critical limitation of conventional approaches in capturing trace cfRNA signals, enabling highly sensitive and accurate detection of low-concentration cfRNA in bodily fluids. It paves a new way for non-invasive molecular diagnostics.^[4]^ The results of high-throughput sequencing also showed that in addition to mRNA, cfRNA also contains a variety of non-coding RNA (ncRNA) types, such as microR-NA (miRNA), piwi-interacting RNAs (piRNAs), tRNA, long non-coding RNA (lncRNA), nucleolar small RNA, *etc*.^[5]^ The broad-spectrum existence and diversity of cfRNAs suggest that cfRNAs, acting as a genetic, epigenetic, and translational regulator, may have great significance and impact on human health by participating in important biological processes, regulating normal growth and development, as well as the occurrence of cancer and disease.^[6]^

Common biomarkers in liquid biopsies include proteins, DNA, RNA, and metabolites. DNA and protein biomarkers play important roles in liquid biopsies. Meanwhile, RNA, which has a special and important status in the central dogma of molecular biology, has been found to act as a possible strongly advantageous biomarker in the occurrence and development of disease by increasing numbers of studies. As a biomarker, cfRNA has several benefits including high sensitivity, high tissue specificity, and low cost of testing, and has drawbacks including instability and the limited detection technology. From the theoretical perspective, RNA differs from DNA for having multiple copies in a single cell and multiple transcriptional regulation forms, and thus can reflect the dynamics of cell states and regulatory processes inside cells. Therefore, large-scale body fluid cfRNA expression profiling can provide information on both genomic differences and transcriptomic dynamic changes, which can be used as direct and accurate biomarkers for non-invasive detection of human health and disease states.^[7,8]^ In addition, the tissue specificity of cfRNA helps overcome the tissue-origin-untraceable defect in circulating tumor DNA (ctDNA) detection, which showed great scientific research value and application prospects. Related studies have shown that cell-free RNAs (cfRNAs) in the blood are more sensitive than cfDNAs in disease detection, and researchers can further identify the tissue source of cfRNAs and evaluate the clinical status of patients through bioinformatics algorithms.^[9,10]^ From the technical perspective, the detection of protein biomarker requires specific antibodies for each marker, and the detection of ctDNA mutations requires ultra-high sequencing depth, both of which are relatively high cost. In contrast, cfRNA sequences can be captured and tracked at high sensitivity and specificity by simple and economical polymerase chain reaction (PCR) techniques.

Cell-free ncRNA is the most common type of cfRNA, including miRNA, piRNA, snRNA, lncRNA, circular RNA (circRNA), *etc*. Due to the protection of cell membrane-like structures and RNA binding proteins, and their own specific structures, cell-free ncRNAs can resist the degradation of RNases in a variety of body fluids, and thus exist stably.^[11]^ This article reviews the characteristics of cell-free ncRNAs, bioinformatics analysis tools, as well as research advances in rheumatic and immune diseases. Finally, the challenges and future research directions of cell-free ncRNAs are discussed.

## A Brief Overview of the Different Types of Cell-free ncRNAs

The evolution of high-throughput sequencing and other technologies has resulted in the identification of a wide range of different classes and sizes of ncRNA, while the biological importance of these ncRNAs also has received much attention. NcRNAs are classified into two broad categories based on length, including small ncRNAs ranging from few to 200 nucleotides (nt) and lncRNAs longer than 200 nt.^[12]^ MiRNAs of approximately 22 nt in size are generally located in intergenic or intronic regions, which are the most abundant class of small ncRNAs known. The human genome encodes over 1, 000 miRNAs, and more than 30% of genes encoding mammalian proteins contain conserved miRNA target sites. Consequently, miRNAs impact a variety of physiological processes in mammals, including epithelial regeneration, cardiac function, ovulation, reproductive health, and cancer progression.^[12]^ These miRNAs function *via* diverse mechanisms that regulate gene expression in the cytoplasm and nucleus and mediate gene silencing at the post-transcriptional level.^[13]^ Moreover, miRNAs can also function as inter-cellular communication molecules by virtue of being secreted in extracellular vesicles or acting as hormones.^[14]^

piRNAs are a novel group of small ncRNA molecules with size of 24–31 nt that frequently bind to members of the piwi family of proteins to fulfil regulatory roles.^[15]^ Contrary to ubiquitous miRNAs, piRNAs are predominantly expressed in the animal gonad.^[16]^ The representative function of piRNAs is to silence transposons at transcriptional and post-transcriptional levels in animal germ cells.^[17]^ The role of the PIWI-piRNA machinery in regulating protein-coding genes in germ cells has apparent gradually surfaced, and the biological merits of the PIWI-piRNA complex have been characterized in germ cells.^[17]^ In fact, there is also a significant amount of piRNA expressed albeit at low levels in somatic tissues, the functional role of which remains to be elucidated.^[18]^

snRNAs are located in the nucleus with the length of ~60–200 nt in length, and constitute conserved non-coding RNAs in eukaryotes.^[19]^ The biosynthesis of most snRNAs involves 3’-terminal nucleolytic cleavage of the nascent transcript by DNA-dependent RNA polymerase II.^[20]^ SnRNAs work with several proteins to form spliceosomes (snRNPs) that are involved in alternative splicing.^[21]^ In eukaryotes, snRNAs play a role in fundamental cellular events such as the regulation of gene expression and ribosomal RNA processing.^[22]^

LncRNAs are a highly diverse group of ncRNAs that account for the largest proportion of the non-coding transcriptome. In general, lncRNAs can be transcribed from virtually every locus in the human genome, and the majority of lncRNAs can be transcribed from intergenic, exonic, or distal proteins coded for in the genome by RNA polymerase II.^[12]^ The diversity of lncRNAs can be reflected in their functions, including transcriptional and translational regulation of neighboring and distal genes. LncRNAs can bind directly to DNA or interact with other RNAs. In addition, lncRNAs can also be used as scaffolds or guides through interactions with proteins, which can facilitate protein co-localization or promote protein-protein interactions.^[14]^ In the cytoplasm, lncRNAs also perform essential functions including the regulation of translation, metabolism and signal transduction.^[23]^

CircRNAs formed by post-splicing of precursor mRNAs (premRNAs) range from 100 nt to over 4 kb in length and are a special type of endogenous ncRNA that can be derived from exons, introns, exon-intron junctions, or intergenic regions of the genome.^[24,25]^ In addition to the effect of back-splicing itself on typical splicing, circRNAs have been reported to modulate gene expression in the nucleus, act as decoys for miR-NAs and proteins, and serve as scaffolds for circRNA-protein complexes. CircRNAs in the nucleus are associated with the regulation of transcription, selective splicing, and chromatin cyclization.^[26]^ While entering the cytoplasm, some circular RNAs can act as competing endogenous RNA (ceRNAs), which are defined as miRNA sponges that bind miRNAs, thus blocking them from binding and repressing target mRNAs.^[27]^ Considering the stable and unique structure of circRNAs, further studies are warranted to enrich their biological functions.

## Characteristics of Cell-free ncRNA

**Figure 1:**
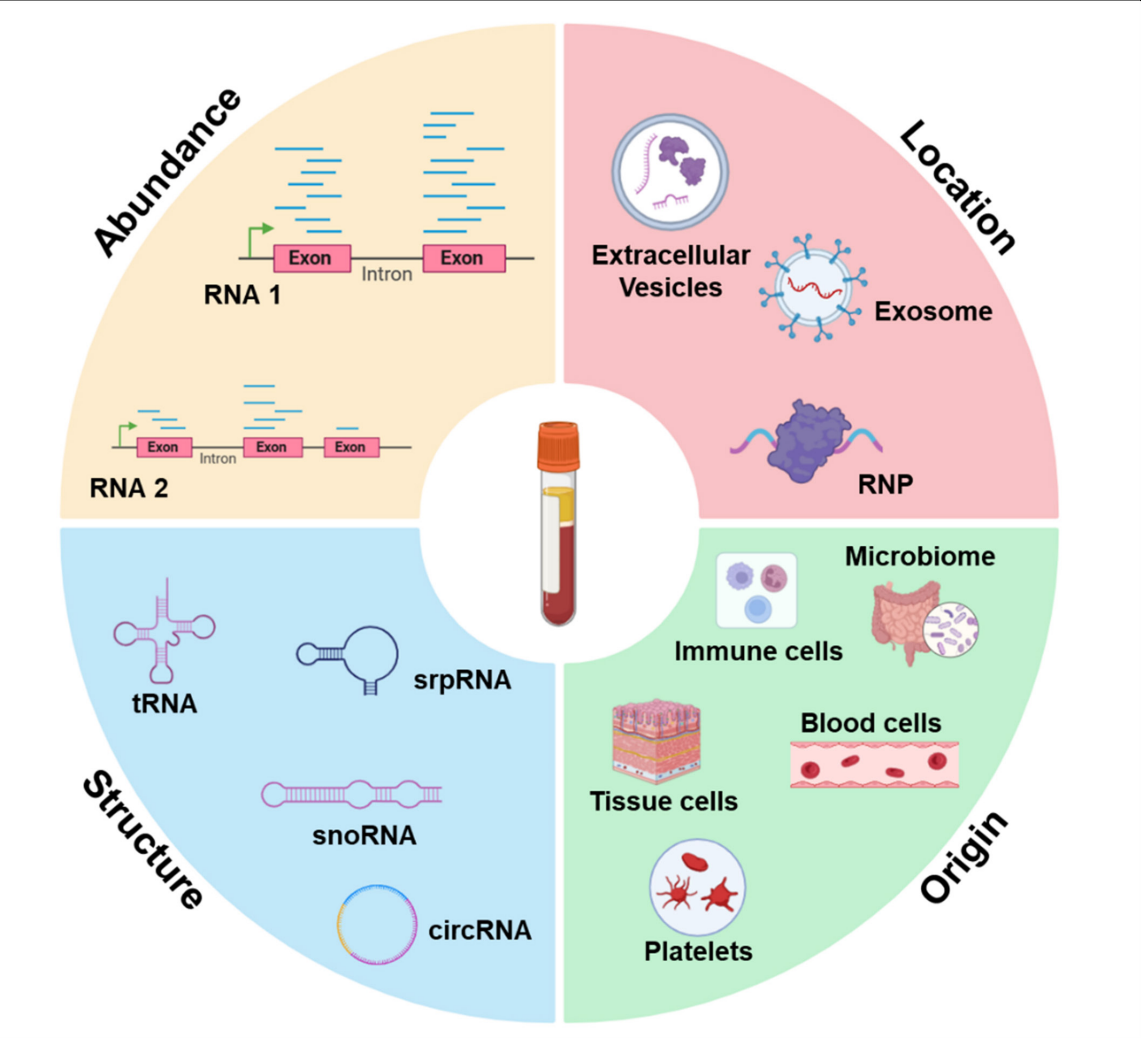
Characteristics of cell-free ncRNAs.

**Location**: RNA is specifically localized in different regions of the cell, starting in the nucleus and then exported to the cytoplasm. Since the extracellular space and body fluids contain a large number of RNases, it was generally considered to be RNAs free. In recent years, an increasing number of studies have found that cell-free ncRNA exists in various encapsulated forms in body fluids, such as extracellular vesicles (EV), including exosomes^[28]^ and microvesicles,^[29]^ as well as lipoprotein particles^[30]^ and argonaute 2 (AGO^2^) protein complexes^[31]^ (Figure 1). EV are heterogeneous structures surrounded by the cell membrane and can be secreted by many types of cells carrying abundant ncRNAs, mainly divided into exosomes and microvesicles, which are shed directly from the endosomal system or originate from the cell membrane, respectively.^[32]^ EVs protect ncRNAs from degradation and are an important carrier and source of cell-free ncRNAs. At the same time, ncRNAs can also be released from cells without EVs and are detected both in the extracellular space and in body fluids as complexes with the protein AGO^2[31,33]^ or high-density lipoproteins (HDLs).^[30,34]^ In addition, platelets formed by the shedding of cytoplasm from megakaryocytes also contain a large number of ncRNAs, which can be used to distinguish early and late stage cancer patients from healthy individuals.^[35]^

**Structure**: The cell-free ncRNAs might be protected from degradation for their specific structures. For example, Zhu *et al*. found that the 5’ region enriched with secondary structure in *SNORD3B-1* can stably exist in plasma, and its abundance can be used as a biomarker for early diagnosis of liver cancer.^[36]^ Tan *et al* identified that the S domain of srpRNA *RN7SL1* was rich in exosomes and showed reliable performance in hepatocellular carcinoma (HCC) diagnosis and prognosis.^[37]^ Moreover, circRNAs are emerging as biomarkers in liquid biopsy for their covalently closed cyclic structure might be responsible for their stability in plasma unlike linear RNAs.^[38]^ Abundant full length tRNAs and tRNA fragments (tRFs) have recently garnered attention as a promising source of biomarkers and a novel mediator in cell-to-cell communication in eukaryotes, tRNAs/ex-tRFs may be protected from degradation by RNases folding into highly stable intermolecular tetramers stabilized by G-quadruplex structures.^[39,40]^

**Origin**: Previous studies have shown that cell-free ncRNA is mainly derived from broken dead cells or live cell signaling mediated by exosomes, such as blood cells, immune cells, microbes, *etc*.^[41, 42, 43]^ Cell-free ncRNA released into the circulation by human tissue cells, cancer cells, which offers an opportunity to detect disease in body fluids such as plasma, since overexpression of pathological tissue specific transcripts may lead to amplification of pathological tissue derived RNA signals in blood. With the development of RNA sequencing technology and bioinformatics methods, cell-free ncRNA shows unique potential in detecting disease and predicting pathological tissue of origin.^[44]^

**Abundance**: Abundance is the most easily detectable and most reflective feature of cell-free ncRNA regulation. Numerous studies have found that abnormal expression of cell-free ncRNA in body fluids can distinguish cancer patients from normal people, which is expected to be used for diagnosis, prediction, and monitoring of diseases. For example, Zhou *et al*. found a set of miRNAs for the diagnosis of hepatitis B virus (HBV)-related hepatocellular carcinoma (HCC).^[45]^ *miR-1*, *miR-133*, and *miR-208* were significantly up-regulated in plasma after myocardial infarction, while *miR-126* was down-regulated in plasma of patients with atherosclerosis.^[46]^ *miR-29* and *miR-124* were significantly up-regulated in the plasma of patients with Alzheimer’s disease, while *miR-9* and *miR-132* were down-regulated in the plasma of patients with Parkinson’s disease.^[47]^ Notably, repeat-derived cfRNA, including simple repeat RNAs and transposable elements RNAs are often present at low levels or undetectable in healthy individuals, but it’s highly enriched in the plasma of tumor patients.^[48]^ In the following sections, aberrant expression of cell-free ncRNAs will be described in more detail in a variety of diseases.

## Bioinformatics Tools and Databases for Cell-free ncRNAs

**Figure 2:**
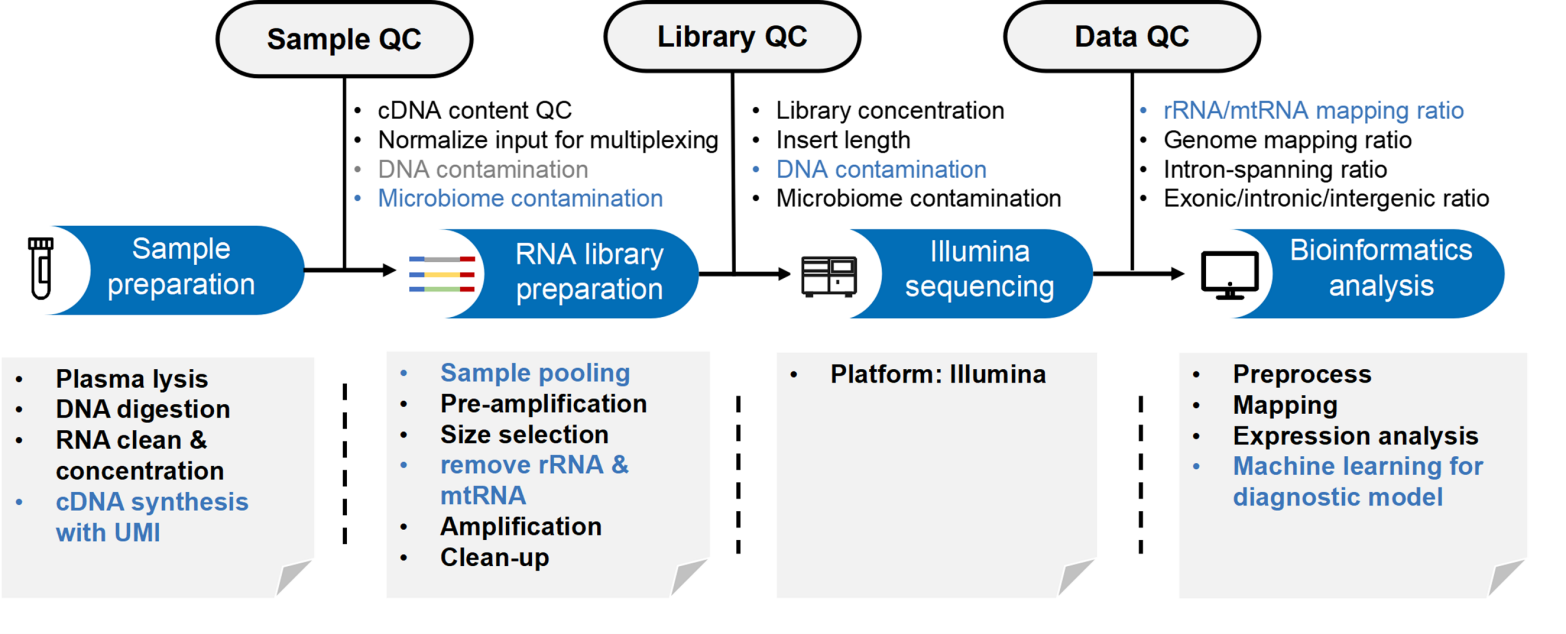
Pipeline of cell-free ncRNA study.

The growing evidence about the presence of cell-free ncRNAs and their role in cell-cell communication and liquid biopsies has outlined the need for suitable processes and tools to collect and analyze these data. Therefore, the pipeline of cell-free ncRNA study includes body fluid samples collection, RNA library preparation, Illumina sequencing and bioinformatics analysis (Figure 2). Through this series of processes, a variety of cell-free ncRNA characteristics can be obtained to provide promising biomarkers for liquid biopsy.

As for bioinformatics tools and databases, exceRpt, a comprehensive analytic platform for cell-free RNA profiling, which has been widely used to process cfRNA-seq data from public datasets.^[49]^ The EVmiRNA database depicts the comprehensive expression profiles of miRNAs in EVs from 462 smRNA sequencing datasets from 17 tissues/diseases, and the biological functions, target genes, pathway regulation and small molecular drug regulations of these miRNAs were analyzed and annotated.^[50]^ liqDB is browsable and interactive database for small RNA-seq profiles in bio-fluids, which provides a user-friendly web interface and useful tool for exploring expression profiles, differential expression analysis, cluster analysis and relevant visualizations of miRNAs from 1607 manually annotated samples.^[51]^ BBCancer is a web-accessible and comprehensive open resource for providing the expression landscape of six type of RNAs, including messenger RNAs (mRNAs), lncRNAs, miRNAs, circRNAs, tRNA derived fragments (tRFRNAs) and piRNAs in 7184 samples including 5040 blood samples such as EVs and circulating tumor cells (CTCs) from normal persons or cancer patients of 15 cancer types.^[52]^ miREV is an online database and tool to uncover potential reference RNAs and biomarkers in small-RNA sequencing data sets from extracellular vesicles enriched samples.^[53]^ exoRBase is a repository of extracellular vesicles long RNAs (exLRs) derived from RNA-seq data analyses in different human fluids. Among them, exoRBase features the integration and visualization of RNA expression profiles, as well as the functional pathway-level changes and the heterogeneity of circulating-EVs origins, which will facilitate the identification of novel exLR signatures from human body fluids and will help discover new circulating biomarkers for the improvement of tumor diagnosis and therapy. EVAtlas database collected small RNA-seq datasets from 2, 030 human EVs, covering 24 conditions and more than 40 diseases. All data were compared by a unified Dynamic reading allocation algorithm (RDAA), and the expression profiles of seven ncRNA types (miRNA, snoRNA, piRNA, snRNA, rRNA, tRNA, and YRNA) were quantified by considering mismatches and multiple mapped read segments.^[54, 55, 56]^ cfOmics is a comprehensive database focusing on extracellular multi-Omics data of multiple diseases, compiling a comprehensive collection of molecular data (including cell-free ncRNAs) from various body fluids, and providing integration, browing, analysis and visualization of multi-omics data^[57]^ (Table 1).

**Table1:** List of bioinformatics analysis tools and databases for cell-free ncRNAs

Bioinformatics Resources	Description	Web Link	Reference
exceRpt	A Comprehensive Analytic Platform for cell-free RNA Profiling	github.gersteinlab.org/exceRpt/genboree.org	[49]
EVmiRNA	a database of miRNA profiling in extracellular vesicles	http://bioinfo.life.hust.edu.cn/EVmiRNA	[50]
LiqDB	a small-RNAseq knowledge discovery database for liquid biopsy studies	http://bioinfo5.ugr.es/Liqdb	[51]
BBCancer	an expression atlas of blood-based biomarkers in the early diagnosis of cancers	http://bbcancer.renlab.org/	[52]
miREV	An Online Database and Tool to Uncover Potential Reference RNAs and Biomarkers in Small-RNA Sequencing Data Sets from Extracellular Vesicles Enriched Samples	https://www.physio.wzw.tum.de/mirev/	[53]
exoRBase	a database of circRNA, lncRNA and mRNA in human blood exosomes	http://www.exoRBase.org	[75]
EVAtlas	a vesicles comprehensive database for ncRNA expression in human extracellular	http://bioinfo.life.hust.edu.cn/EVAtlas	[55]
NPInter	a ncRNAs database and that other collects biomolecules information on the interactions between	http://bigdata.ibp.ac.cn/npinter5/	[54]
ncRNADrug	a database of ncRNAs related to drug resistance and drug targeting	http://www.jianglab.cn/ncRNADrug	[56]
cfOmics	cfOmics: a cell-free multi-Omics database for diseases	https://cfomics.ncRNAlab.org/	[57]

These tools and websites also have certain limitations. For instance, exceRpt is an analysis platform specifically developed for small RNA sequencing, but there is a lack of tools suitable for analyzing cfRNA in long RNA sequencing data.^[49]^ Regarding EVmiRNA, this database primarily focuses on cancers such as prostate cancer and breast cancer, but does not include non-cancer-related data.^[50]^ For LiqDB, although it demonstrates satisfactory integration performance for samples processed with different library preparation protocols, its effectiveness in large-scale sample cohorts may require further validation.^[51]^ Regarding BBCancer, while this database provides valuable insights into the abundance and differential expression profiles of diverse ncRNAs in cancer contexts, it currently lacks dedicated modules for functional enrichment analysis to elucidate the biological significance of these RNAs.^[52]^ For miREV, it similarly lacks modules for functional enrichment analysis of these miRNAs, limiting its utility for in-depth biological interpretation.^[53]^ An additional limitation shared by these tools/databases is the difficulty in maintaining consistent or timely updates, necessitating the continual development of novel tools or databases to meet evolving research demands.

## Compendium of Cell-free ncRNA Biomarkers in Rheumatic and Immune Diseases

Rheumatic and immune diseases mainly include Rheumatoid arthritis (RA), systemic lupus erythematosus (SLE), Sjögren’s syndrome (SS) and systemic sclerosis (SSc), among others. As for laboratory tests or clinical practice, each rheumatic and immune disease has its own diagnosis method, but there is no generally accepted diagnostic standard. For example, Laboratory tests, such as Complement (C3, C4) and antibodies (ANA, anti-dsDNA, antiphospholipid, *etc*.) have although been demonstrated to have potential as SLE diagnostic biomarkers, but none of them can accurately diagnose SLE. In clinical practice, the diagnosis of SLE is mainly according to the revised American College of Rheumatology (ACR) classification criteria, which is made based on clinical manifestations and laboratory tests.^[58]^ Aberrant expression of various cell-free ncRNA has been found to distinguish people with rheumatic and immune diseases from healthy individuals (Table 2).

## RA

RA is an autoimmune disease characterized by synovial inflammation. Recent studies have shown that the levels of miRNA-21 and miRNA-146a in the peripheral blood serum of RA patients are significantly elevated. miRNA-146a exhibits a significant positive correlation with the DAS-28 score and clinical manifestations in RA patients, including morning stiffness, joint tenderness, and swelling.^[59]^ Another study demonstrated that miR-146b-3p is upregulated in the serum and synovial tissues of RA patients. Silencing miR-146b-3p attenuated TNF-α-induced proliferation and migration of MH7A cells, suggesting that miR-146b-3p not only serves as a diagnostic biomarker for RA but also plays a crucial pro-inflammatory and pro-proliferative role.^[60]^ A study comprising 76 RA patients, 30 SLE patients, 32 SS patients, and 36 healthy controls demonstrated that plasma miR-22–3p and let-7a-5p could effectively discriminate RA patients from healthy individuals, with AUC values reaching 0.812 and 0.832, respectively.^[61]^ Moreover, miR-22–3p and let-7a-5p exhibited strong discriminatory capacity to differentiate RA from other rheumatic diseases, including SLE and SS, with AUC values consistently exceeding 0.75.^[61]^ In addition, the over-expression of *let-7a-5p, let-7b-5p, let-7 d-5p, let-7f-5p, let-7 g-5p, let-7i-5p, miR-128–3p, miR-25–3p* can be used as biomarkers for diagnose of RA.^[62]^

**Table 2:** A compendium of cell-free ncRNAs with potential diagnostic or prognostic role for patients with immune mediated diseases*

RNA Species	Disease species	Name	Source	Usage	Up/Down^△^	Reference
miRNA	Rheumatoid arthritis	miR-126-3p, let-7 d-5p, miR-221-3p	Serum	Diagnosis	Up	[76]
miRNA	Rheumatoid arthritis	miR-125a, miR-125b	Plasma	Prognosis	Up	[77]
miRNA	Rheumatoid arthritis	miR-125a-5p, miR-24	Plasma	Diagnosis	Up	[78]
miRNA	Rheumatoid arthritis	miR-146a-5p	Plasma	Diagnosis	Up	[79]
miRNA	Rheumatoid arthritis	let-7a-5p, let-7b-5p, let-7 d-5p, let-7f-5p, let-7 g-5p, let-7i-5p, miR-128-3p, miR-25-3p	Exosomes	Diagnosis	Up	[77]
miRNA	Rheumatoid arthritis	miR-22-3p and let-7a-5p	plasma	Diagnosis	Up	[61]
miRNA	Systemic lupus erythematosus	miR-551b, miR-448	Serum	Diagnosis	Up	[66]
miRNA	Systemic lupus erythematosus	miR-124	Serum	Diagnosis	Down	[80]
miRNA	Systemic lupus erythematosus	miR-125b, miR-101, miR-375	Plasma	Prognosis	Down	[81]
miRNA	Systemic lupus erythematosus	miRNA-21	Serum	Diagnosis	Up	[67]
lncRNA	Systemic lupus erythematosus	GAS5	plasma	Diagnosis	Down	[63]
lncRNA	Sjögren’s syndrome	lnc-DC	plasma	Diagnosis	Up	[69]
lncRNA	Sjögren’s syndrome	miR-17-5p and let-7i-5p	Salivary	Diagnosis	Down	[70]
miRNA	Systemic sclerosis	miR-214	plasma	Diagnosis	Down	[72]
miRNA	Systemic sclerosis	miR-20a-5p, miR-21-5p	plasma	Diagnosis	Down, up	[73]

* This table lists the representative cell-free ncRNA biomarkers for pregnancy-related diseases (AUC > 0.8). ^△^Up: Higher abundance in pregnancy-related diseases patient than healthy control; Down: lower abundance in pregnancy-related diseases patient than healthy control.

## SLE

SLE is a clinically heterogeneous disease caused by dysregulation of the immune system and loss of self-tolerance. Emerging evidence highlights the diagnostic potential of lncRNAs in SLE. A study revealed that the expression of GAS5 and lnc7074 was significantly down-regulated in SLE patients compared to healthy subjects, whereas lnc0640 and lnc5150 exhibited elevated levels. These differentially expressed lncRNAs demonstrate high diagnostic accuracy for SLE and exhibit specificity in distinguishing SLE from RA.^[63]^ Another study detected 2, 353 dysregulated lncRNAs in the plasma of SLE patients, among which YPEL4 was associated with the FcγR pathway.^[64]^ YPEL4 may contribute to SLE pathogenesis by stimulating immune cells to release inflammatory mediators.^[65]^ In serum, *miR-551b*, *miR-448* and *miRNA-21* were up-regulated in patients with SLE, while *miR-124* was down-regulated.^[66,67]^

## SS

Primary SS (pSS) is an autoimmune disease characterized by dry eyes and dry mouth as its predominant clinical manifestations.^[68]^ A study involving 109 healthy controls (HC), 50 SLE patients, 50 RA patients, and 127 pSS patients demonstrated that lnc-DC levels were significantly higher in pSS patients compared to HC, SLE, and RA patients. The AUC value of lnc-DC for distinguishing pSS from healthy individuals reached 0.80.^[69]^ The combination of lnc-DC with anti-SSA and anti-SSB antibodies could further improve the diagnostic efficacy for pSS, achieving an AUC value of 0.84.^[69]^ Salivary miR-17–5p and let-7i-5p also demonstrated high diagnostic performance for pSS, with AUC values reaching 0.87 and 0.91134, respectively. Furthermore, these miRNAs showed significant correlations with salivary flow rate and histopathological features in patients.^[70]^

## SSc

SSc is an autoimmune disease characterized by vascular pathology, chronic inflammation, and fibrosis, primarily affecting connective tissues throughout the body.^[71]^ A study involving 40 female SSc patients and 14 healthy female controls revealed that plasma miR-214 expression was significantly downregulated in SSc patients compared with controls. Notably, miR-214 demonstrated excellent discriminative capacity for SSc identification, with an AUC of 0.80.^[72]^ Furthermore, the combination of plasma miR-20a-5p and miR-21–5p demonstrated significant diagnostic potential for SSc-associated pulmonary arterial hypertension (SSc-PAH), achieving an AUC value of 0.83.^[73]^

## Conclusions and Perspectives

ncRNA includes small RNA and long ncRNA, which are called “dark matter” or “junk RNA” in the genome because they cannot encode proteins. Recent studies have shown that ncRNAs play important roles in many life processes. Cell-free ncRNAs in serum, plasma, saliva, urine, and other body fluids have been implicated in various pathological conditions. Cell-free ncRNAs circulate in body fluids with a highly stable extracellular form due to their secondary structures or their associations with biological macromolecules such as proteins. Given that body fluids are more readily accessible than tissue samples, cell-free ncRNAs are frequently employed as biomarkers in liquid biopsy applications. Nevertheless, there are still many drawbacks and challenges in moving this field forward and implementing clinical applications: Firstly, cfRNA itself is highly fragmented, heterogeneous, and has a low signal-to-noise ratio (the proportion of lesion-derived cfRNA is low), presenting significant challenges for its sensitive detection and clinical application. Secondly, RNA is prone to degradation, posing significant challenges for sample preservation in clinical application. Thirdly, the lack of standardized analytical strategies for cell-free non-coding RNA (cf-ncRNA) hinders comparability across different studies. Lastly, the clinical application of cfRNA requires validation through large-scale, multi-center studies with diverse sample cohorts. There are currently a multitude of methods to isolate RNA from biological fluids, and various protocols and kits affect downstream sequencing and PCR results, and the potential bias of these methods needs to be considered carefully. As well, we are required to overcome technical challenges to consider how to isolate pure cell-free ncRNAs. Future research ought to elucidate the sources, mechanisms of export and uptake of ncRNAs in each of the biofluids evaluated. We can further investigate the biological functions and clinical significance of cell-free ncRNAs by knocking down or overexpressing these RNAs in their cells or animal tissues of origin. The storage of cell-free ncRNA and its verification in multi-center populations with large sample sizes are also challenges in the process of its clinical application. It is also important to continue to develop computational techniques and tools to fully exploit and widely utilize cfRNA data. Based on cell type-associated signature computational methods, cfRNA can trace its cellular origins and characterize cellular pathological changes during disease development.^[74]^ This positions cfRNA as a non-invasive biomarker for monitoring disease progression and *in vivo* drug responses. For exosome-derived cfRNA, further investigation can reveal whether its active secretion mechanism correlates with rheumatic and immune diseases onset/progression.
